# The diminishing returns of atovaquone-proguanil for elimination of *Plasmodium falciparum* malaria: modelling mass drug administration and treatment

**DOI:** 10.1186/1475-2875-13-380

**Published:** 2014-09-24

**Authors:** Richard J Maude, Chea Nguon, Arjen M Dondorp, Lisa J White, Nicholas J White

**Affiliations:** Mahidol-Oxford Tropical Medicine Research Unit, Faculty of Tropical Medicine, Mahidol University, Bangkok, Thailand; Centre for Tropical Medicine, Nuffield Department of Medicine, University of Oxford, Old Road, Oxford, OX3 7LJ UK; National Centre for Parasitology, Entomology and Malaria Control, Phnom Penh, Cambodia

**Keywords:** Mathematical, Model, Falciparum, Malaria, Atovaquone, Artemisinin, Dihydroartemisinin, Resistance

## Abstract

**Background:**

Artemisinin resistance is a major threat to current efforts to eliminate *Plasmodium falciparum* malaria which rely heavily on the continuing efficacy of artemisinin combination therapy (ACT). It has been suggested that ACT should not be used in mass drug administration (MDA) in areas where artemisinin-resistant *P. falciparum* is prevalent, and that atovaquone-proguanil (A-P) might be a preferable alternative. However, a single point mutation in the cytochrome b gene confers high level resistance to atovaquone, and such mutant parasites arise frequently during treatment making A-P a vulnerable tool for elimination.

**Methods:**

A deterministic, population level, mathematical model was developed based on data from Cambodia to explore the possible effects of large-scale use of A-P compared to dihydroartemisinin-piperaquine ACT for mass drug administration and/or treatment of *P. falciparum* malaria, with and without adjunctive primaquine (PQ) and long-lasting insecticide-treated bed nets (LLIN). The aim was local elimination.

**Results:**

The model showed the initial efficacy of ACT and A-P for MDA to be similar. However, each round of A-P MDA resulted in rapid acquisition and spread of atovaquone resistance. Even a single round of MDA could compromise efficacy sufficient to preclude its use for treatment or prophylaxis. A switch to A-P for treatment of symptomatic episodes resulted in a complete loss of efficacy in the population within four to five years of its introduction. The impact of MDA was temporary and a combination of maintained high coverage with ACT treatment for symptomatic individuals and LLIN was necessary for elimination.

**Conclusion:**

For malaria elimination, A-P for MDA or treatment of symptomatic cases should be avoided. A combined strategy of high coverage with ACT for treatment of symptomatic episodes, LLIN and ACT + P MDA would be preferable.

**Electronic supplementary material:**

The online version of this article (doi:10.1186/1475-2875-13-380) contains supplementary material, which is available to authorized users.

## Background

Efforts are underway to eliminate *Plasmodium falciparum* malaria in many malaria-endemic countries [1]. A major threat to these efforts is the recent identification of artemisinin resistance in Cambodia [2], Thailand [3], Myanmar [4], and Vietnam [5]. Artemisinin combination therapy (ACT) is first-line treatment for malaria worldwide and thus an important component of strategies for malaria elimination.

Mathematical modelling predicts that by using ACT at high coverage, as part of a combined strategy, artemisinin-resistant malaria can be eliminated. To do so would require elimination of all malaria in an area as the last few infections to be cleared are most likely to be resistant [6]. Atovaquone-proguanil (A-P) has been proposed as an alternative anti-malarial to ACT to reduce selective pressure for the spread of artemisinin resistance, and has been deployed as first-line treatment in western Cambodia and adjacent parts of Thailand. A-P is also being considered as a candidate for mass drug administration (MDA), rather than treatment, in Cambodia [7]. Using A-P for MDA in the presence of artemisinin resistance has the advantage of not adding additional artemisinin drug pressure on the parasite population. A major drawback of A-P is the frequent emergence of mutations conferring high level atovaquone resistance, rendering the drug dangerously ineffective [8]. A single point mutation in the cytochrome b (*cyt b)* gene, which occurs at a viable frequency around 1:10^12^ parasites, encodes high-level resistance.

A mathematical model was developed to explore the possible effects of large-scale use of A-P compared to dihydroartemisinin-piperaquine ACT for MDA and/or treatment of *P. falciparum* malaria, with and without adjunctive primaquine (PQ) and long-lasting insecticide-treated bed nets (LLIN), with the aim of local elimination.

## Methods

**Figure 1:**
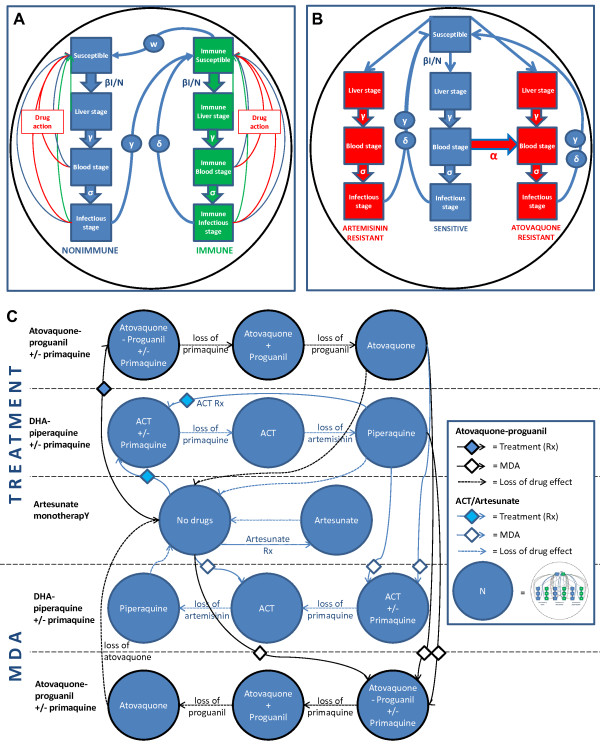
**Structure of the mathematical model. A** shows the basic model unit with parasite life cycle stages in the human host, anti-malarial drug action and immunity. ‘Blood stage’ refers to individuals with asexual stage parasites in the peripheral blood but no gametocytes and ‘infectious stage’ is individuals with gametocytes. **B** shows the unit in **A** repeated three times to track parasites resistant to artemisinins and atovaquone with appearance of novel atovaquone-resistant infections indicated by alpha. **C** shows multiple repetitions of **B** to simulate treatment and/or MDA with atovaquone-proguanil, ACT and/or primaquine.

A deterministic, population level, mathematical model of *P. falciparum* malaria transmission and treatment in low transmission settings was developed. It was based on a previously published modelling framework for elimination of artemisinin-resistant *P. falciparum* malaria in Cambodia [9] and was solved numerically using Berkeley Madonnna™ (CA, USA). The model includes the effects of anti-malarial treatment at different stages of the parasite life cycle, atovaquone and artemisinin resistance, seasonally varying transmission intensity, symptomatic and asymptomatic infections and immunity. The updated model structure is shown diagrammatically in Figure 1. Model assumptions are listed in Additional file 1: Table S1, the parameter values and their sources in Additional file 2: Table S2. Treatment and MDA in the model were with either three days of once daily dihydroartemisinin-piperaquine (referred to as ‘ACT’ throughout the text) or three days of once daily A-P. The model was designed to predict the efficacy of interventions in low transmission settings.

Western Cambodia was considered in detail as a case study for elimination of artemisinin-resistant malaria but the findings are generalizable to any low transmission setting with a median multiplicity of infection of 1 and seasonal variation in transmission intensity. There have been various estimates of parasite prevalence (proportion of population with parasites of any stage in the peripheral blood) in parts of Cambodia using microscopy, spleen rates and polymerase chain reaction. In the absence of a consensus for western Cambodia, in the model this was set at a mean of 15%. To approximate the seasonal variation in parasite prevalence in western Cambodia, the model was fitted using the least squares method to national malaria surveillance data from the Cambodia National Malaria Control Programme, as described elsewhere [9]. These data and derived estimates of total malaria burden over time in Cambodia will be published separately. The proportion of infections that were artemisinin-resistant in 2013 in western Cambodia was unknown, although studies are underway in some areas that will provide estimates of this. In the model, in western Cambodia 10% of infections were assumed to be artemisinin resistant. Atovaquone resistance was assumed to be absent prior to the introduction of A-P and a fitness cost of atovaquone resistance was modelled as a reduction in contribution to transmission by the resistant parasites of 0-9% [10]. Symptomatic and asymptomatic infections were assumed to have different rates of acquisition of resistance mutations to atovaquone proportional to the relative median parasite biomass for individuals in each group. Coverage with anti-malarials was the same for ACT and A-P wherever their efficacy was compared.

The model was used to answer the following questions for a reduction in proportion infected with, and ideally elimination of, *P. falciparum* malaria in low transmission settings: 1) for MDA: a) what is the relative efficacy of A-P *versus* ACT?; b) what is the additional impact of adjunctive low dose PQ?; c) what are the optimal timing and coverage?; d) how does the impact of LLIN compare with that of MDA?; and, e) how does the impact of MDA vary with transmission intensity? 2) For treatment of symptomatic patients: what is the relative efficacy of A-P *versus* ACT in the long-term? 3) What interventions are required to eliminate malaria?

## Results

### Atovaquone-proguanil *versus*artemisinin combination therapy for mass drug administration

**Figure 2:**
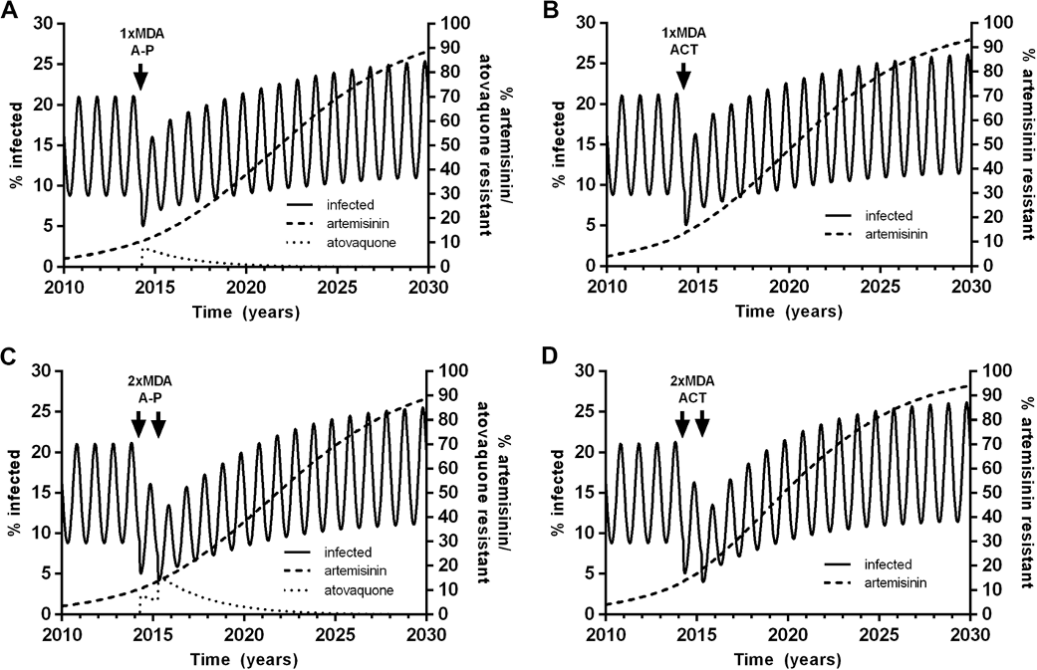
**Effect of MDA on parasite prevalence and antimalarial resistance.** Effects of **A** atovaquone-proguanil and **B** ACT for a single round of MDA on the % of the population infected with *P. falciparum* over time and the proportions of infections which are resistant to artemisinin and/or atovaquone. **C** and **D** show the effects of two rounds of MDA one year apart. MDA was at 95% coverage in western Cambodia.

In the model, A-P and ACT had very similar efficacies in reducing parasite prevalence when used for MDA, although this effect was transient (Table 1). The efficacy of a single round of ACT MDA was slightly less than that of A-P at the same coverage (Figure 2) because 10% of infections were artemisinin resistant at the time of the MDA and because of the causal prophylactic effect of A-P against liver stage parasites [11]. During one round of A-P MDA, *de novo* atovaquone resistance appeared and increased to 8.4% of infections by the end of the MDA and had fallen to 5.2% one year later. Because of this, the efficacy of a second round of MDA with A-P one year after the first was diminished with a subsequent nadir parasite prevalence of 3.9%. With two successive annual rounds of MDA with A-P, the proportion resistant to atovaquone increased further to over 19% (Figure 2C). In contrast, the pre-existing artemisinin resistance increased very little with a round of ACT MDA, thus the efficacy of a second round of ACT MDA was relatively greater with ACT (Figure 2D) than with A-P. An alternative strategy of alternating annual rounds of A-P and ACT MDA for four years, i.e. A-P, ACT, A-P then ACT, resulted in a lower peak in atovaquone resistance of 17% and a nadir parasite prevalence of 2.7%. A-P, ACT, ACT then A-P resulted in a peak in atovaquone resistance of 15% and nadir parasite prevalence of 2.8%.

**Table 1:** **The modelled nadir and % reductions in pararasite prevalence (% infected** ***with P. falciparum*** ) in the year following one (*1) or two (*2) annual rounds of MDA using atovaquone-proguanil or ACT +/− primaquine in western Cambodia

MDA strategy used	Nadir parasite prevalence (%)	% decrease in nadir with MDA
Nil	8.9	
Atovaquone-proguanil*1	4.8	45.6
(Atovaquone-proguanil + primaquine)*1	4.6	48.7
Atovaquone-proguanil*2	3.9	56.2
(Atovaquone-proguanil + primaquine)*2	3.5	60.4
ACT*1	5.1	43.1
(ACT + primaquine)*1	4.7	47.7
ACT*2	4.0	55.4
(ACT + primaquine)*2	3.5	60.9

In the examples shown, the MDA was done at the time of maximum efficacy (1–2 months before nadir parasite prevalence).

Due to the uncertainty in the model parameters determining the *de novo* acquisition of atovaquone resistance, a sensitivity analysis was performed to assess their effect on the peak proportion of infections resistant to atovaquone after one MDA with A-P. Three parameters were varied: para_sym:asym, the relative median parasite burdens in symptomatic *versus* asymptomatic individuals, from 100,000:1 to 1:1 with peak % resistant from 8.4 to 13.8%; acq_rvdvg_, the rate of atovaquone resistance arising during treatment with A-P, from 1/1,000 to 1/10 days^−1^ with peak % resistant from 8.3 to 10.3%; and acq_rvdv,_ the rate of atovaquone resistance arising during treatment with the atovaquone pharmacokinetic tail of A-P, from 1/10 to 1/0.5 days^−1^ with peak % resistant from 2.3 to 9.9%. Varying all three parameters simultaneously resulted in a range of peak % resistant to atovaquone from 2.1 to 18.8%. Varying the fitness cost of atovaquone resistance (cost_v_) from 0 to 9% made minimal difference to the results except that the greater the fitness cost, the faster the rate of decay in proportion of atovaquone-resistant infections following an MDA. With cost = 0% there was no decay and proportion resistant remained the same in the years following the MDA.

Adding PQ to one round of MDA with ACT or A-P resulted in virtually the same overall decrease in parasite prevalence with both drug choices. When two rounds of MDA with additional PQ were modelled, the drop in parasite prevalence was slightly greater with ACT than with A-P.

### Optimal timing of mass drug administration

**Figure 3:**
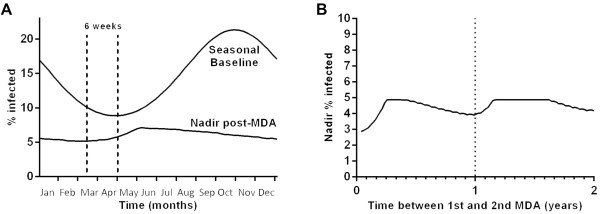
**Effect of varying the timing of atovaquone-proguanil MDA on nadir parasite prevalence. A**. Timing of a single round of MDA relative to the nadir in the seasonal baseline. **B**. Varying the time between two episodes of MDA.

The maximal reduction in parasite prevalence was achieved with MDA one to two months before the seasonal nadir in parasite prevalence (Figure 3A). The optimal timing of a second round of MDA to maximize reduction in parasite prevalence was as soon as possible after the first, as long as this was before the seasonal nadir in parasite prevalence i.e. within one to two months of commencing the first round. With this aggressive strategy, a nadir parasite prevalence of 2.9% could be achieved for A-P and 3.0% for ACT with two rounds of MDA. However, with A-P, atovaquone resistance increased more rapidly to almost 24% of infections. If the second round of MDA was after the seasonal nadir, then the optimal time for it was in the next transmission season one to two months before the next seasonal nadir (Figure 3B). Results were similar for A-P and for ACT.

### Coverage with mass drug administration

**Figure 4:**
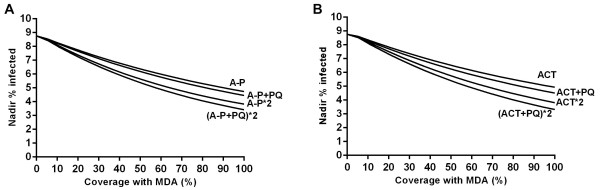
**Effect of increasing coverage with MDA on the nadir parasite prevalence achieved. A**: atovaquone-proguanil **B**: ACT. Lines are shown for one or two annual rounds of MDA with and without primaquine.

The efficacy of MDA was non-linearly related to population coverage (Figure 4). The relative increase in efficacy decreased with increasing coverage for all MDA regimens modelled.

### Bed nets *versus*mass drug administration

**Figure 5:**
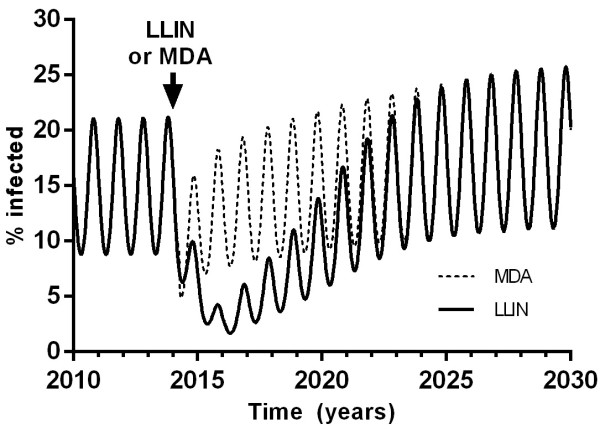
**Efficacy of LLINs** ***versus*** **MDA.** In this example, the LLINs were distributed on one occasion to 60% of the population and the MDA was a single round with atovaquone-proguanil with coverage 95%, both in 2014.

The relative efficacy of introduction of LLINs (assuming 30% reduction in force of infection and half-life of two years) on a single occasion was compared to a single round of MDA (Figure 5). Although the initial effect of reducing parasite prevalence was greater for MDA, the LLINs continued to reduce transmission for longer due to their long half-life and the overall effect was a greater eventual fall in parasite prevalence with LLINs. Parasite prevalence took longer to return to pre-intervention levels after LLINs than after MDA. Two rounds of MDA with 95% coverage one year apart had a similar magnitude and duration of efficacy to a single distribution of bed nets with 25% population coverage. Of course these benefits of LLINs are critically dependent on their reduction in the force of infection and sustained efficacy (i.e. no insecticide resistance).

### Efficacy of mass drug administration in different transmission settings

**Figure 6:**
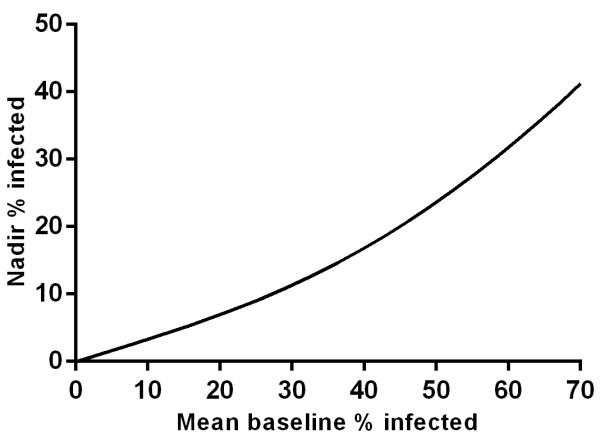
**Efficacy of MDA in different transmission settings.** Nadir % infected following a single round of atovaquone-proguanil MDA in settings of different transmission intensity using baseline % infected with *P. falciparum* as a proxy.

The modelled efficacy of MDA was non-linearly related to transmission intensity in that in settings with higher baseline parasite prevalence, the efficacy of MDA was relatively lower (Figure 6).

### Switching treatment to atovaquone-proguanil

**Figure 7:**
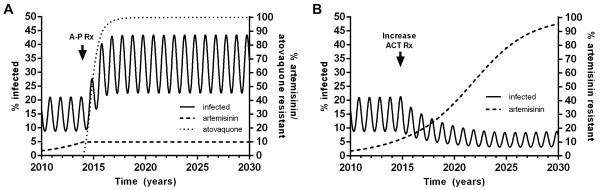
**Effect of treatment on the spread of resistance. A**: Switch of first-line treatment in 2014 from ACT to the same coverage of 72% with atovaquone-proguanil and the resulting spread of atovaquone resistance. **B**: increase of ACT coverage in 2014 to 95% and artemisinin resistance.

When treatment of symptomatic episodes in the model was switched from ACT to A-P at the same coverage of 72%, atovaquone resistance arose rapidly and spread, resulting in an increase in malaria prevalence in the population within one year of its introduction and complete loss of A-P efficacy (>99% atovaquone resistance) as a treatment within four years (Figure 7).

These results were robust to a sensitivity analysis for parameters determining the rate of acquisition of atovaquone resistance. Variation of acq_rvdvg_ from 1/1,000 to 1/10 days^−1^ made no noticeable difference to the results. Varying acq_rvdv_ from 1/0.5 to 1/10 days^−1^, resulted in a small change in the time to >99% atovaquone resistance from four to five years.

### Combined strategies

**Figure 8:**
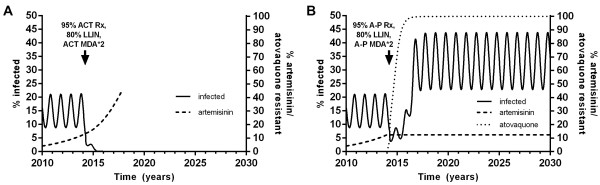
**Combined strategies to achieve malaria elimination. A**: Optimal strategy implemented in 2014 of MDA with 2 annual rounds of ACT + P at 95% coverage, 95% coverage with ACT for treatment maintained until elimination and 80% coverage with LLIN. **B**: The same strategy as **A** using A-P in place of ACT.

It was only possible to eliminate malaria in the model when multiple strategies were used. High coverages with both ACT and LLIN were essential to achieve elimination whereas MDA was not. The most effective strategy was multiple rounds of MDA with ACT + P, high coverage with ACT for treatment of symptomatic cases maintained until elimination, and long-lasting insecticide-treated bed nets (Figure 8A). With two rounds of MDA 1 year apart at 95% coverage and LLIN at 80% coverage, elimination was achievable with maintained treatment coverage for symptomatic cases of >86% in under 4 years. Without MDA, >93% treatment coverage was required for elimination. Where A-P was used for MDA and treatment in place of ACT in a combined strategy with LLIN, elimination was not possible due to rapidly spreading atovaquone resistance (Figure 8B).

## Discussion

The modelled initial efficacy of A-P and dihydroartemisinin-piperaquine ACT for MDA was similar. However, the rapid acquisition and spread of resistance to atovaquone caused the efficacy of A-P to be compromised within a short timeframe. With each round of MDA, the proportion of infections resistant to atovaquone increased greatly, resulting in diminishing returns for a strategy of this type. This did not occur with artemisinin resistance, which spread very little during a round of MDA. This is partly because of the relatively mild phenotype of artemisinin resistance providing a relatively smaller survival advantage than for atovaquone and because of the much higher frequency of spontaneous appearance of novel resistance-conferring mutations for atovaquone than is thought to be the case for artemisinin resistance [12, 13].

With the introduction of A-P for first-line treatment in the model, atovaquone resistance rapidly appeared and spread through the population, resulting in an increase in malaria prevalence in the population within one year of its introduction and complete loss of efficacy as a treatment in four to five years. This is in agreement with the rapid spread of atovaquone resistance shortly after the switch to A-P introduction in the field in western Cambodia and is additional evidence that A-P should not be used for this purpose.

The modelling suggests that use of A-P for MDA is questionable, despite the advantage of avoiding additional artemisinin drug pressure. The modelling predicts that the contribution to spread of artemisinin resistance from ACT MDA is minimal. With repeated rounds of ACT MDA the effect on resistance spread is cumulative, although still small. If A-P were to be used, the proportion of infections resistant to atovaquone rose to around 10% of infections with most parameter combinations, following a single round of MDA. The WHO recommends a change of anti-malarial drug in a national malaria treatment policy if treatment failure proportion is ≥10% [14]. This would preclude the use of A-P for treatment or prophylaxis following even a single round of A-P MDA. Additionally, A-P can be much more expensive than ACT, although drug cost is only one component of the programme cost of MDA. A combined strategy, including ACT MDA and LLINs and high coverage with ACT for treatment of symptomatic episodes would be a better option for Cambodia. The modelling suggests that the efficacy and efficiency of the MDA used can be optimized by timing it one to two months before the seasonal nadir in parasite prevalence and employing repeated episodes around one year apart. Two rounds of MDA in rapid succession before the seasonal nadir in transmission are predicted to be even more effective, but may be difficult to implement in the field. An optimal coverage level is difficult to define but the modelling indicates there is little benefit to be gained from small increases in already high coverage as this tails off in a non-linear fashion. Very high levels of coverage are difficult to attain and incur proportionately higher costs. A compromise between cost and coverage would seem reasonable. Low dose PQ added modestly to the efficacy of MDA with either A-P or ACT and should be considered, particularly in areas threatened by artemisinin resistance and areas attempting elimination [15]. However, achievable coverage may be limited by its side-effect of haemolysis in glucose-6-phosphate dehydrogenase deficient individuals.

## Conclusions

For malaria elimination, A-P for MDA or treatment of symptomatic cases should be avoided because of the rapid appearance and spread of atovaquone resistance. A combined strategy of ACT + P MDA, LLINs and high coverage with ACT for treatment of symptomatic episodes would be preferable.

## Electronic supplementary material

Additional file 1: Table S1: Assumptions. (PDF 207 KB)

Additional file 2: Table S2: Parameters. (PDF 308 KB)

## Abbreviations

ACT: Artemisinin combination therapy
A-P: Atovaquone-proguanil
LLINs: Long-lasting insecticide-treated bed nets
MDA: Mass drug administration
PQ: Primaquine.
