# Supplementary material for: The diminishing returns of atovaquone-proguanil for elimination of Plasmodium falciparum malaria: modelling mass drug administration and treatment
Source: Malar J. 2014 Sep 24;13:380. doi: 10.1186/1475-2875-13-380 (PMC4192368; doi:10.1186/1475-2875-13-380)
Supplement: Supplementary file 1 — Additional file 1: Table S1: Assumptions. (PDF 207 KB) [file 12936_2014_3549_MOESM1_ESM.pdf]

## Assumptions

| | Assumption | Justification for making the assumption | Reasoning for likely effect on efficacy of interventions if assumption is true |
|---|---|---|---|
| **Likely to *decrease* efficacy of interventions** | No mortality due to malaria. | The proportion of malaria infections which are fatal in this region is around 0.6% [1], a small proportion of overall infections. | Those people with resistant infections are less likely to respond to treatment and therefore more likely to die, thus removing them from the transmitting population. |
| | Artesunate monotherapy was the only available effective treatment in Cambodia before 2000. | Although co-blistered artesunate and mefloquine has been the official first-line drug since 2000, in 2002 a wide range of treatments were available over the counter in Cambodia. Many received artesunate monotherapy and most of the other treatments were inadequate (resistant parasites/wrong dose/wrong duration) to cure infection.[2] | Artesunate would be less likely to cure artemisinin-resistant infections than non-artemisinin drugs, if available. If significant amounts of ACT had been available, this would decrease the baseline parasite prevalence. |
| | No treatment of nonmalarial fever with antimalarials. | Presumptive treatment of malaria is not standard practice in Cambodia and is discouraged worldwide.[3]<br><br>There were no data available with which to parameterise such a model for Cambodia.<br><br>To maintain simplicity. | Some people with incidental asymptomatic malaria infection would also receive treatment. |

| | | | |
|---|---|---|---|
| **Likely to *increase* efficacy of interventions** | There is no pre-existing atovaquone resistance prior to its' use in an intervention. | Expert opinion in the absence of data | If there was pre-existing atovaquone resistance then interventions using atovaquone-proguanil would be less effective. |
| | | To maintain simplicity. | |
| | No spatial heterogeneity | A non-spatial framework was chosen to maintain flexibility and efficiency to rapidly explore a wide range of scenarios in accordance with the aims of this study. | Infection in high transmission areas is harder to eliminate therefore overall elimination of malaria would take longer. |
| | | In the absence of data about many spatially heterogeneous parameters (seasonal variation in parasite prevalence, coverage with interventions, geographical extent of each village, etc.) we felt their incorporation at this stage was premature. | |
| | | We are in the ongoing process of gathering detailed data to develop a spatial model based on the framework presented here. | |
| | No population migration. | To maintain simplicity. | In-migration of infected individuals would slow the reduction of malaria, possibly precluding its elimination, and could introduce new resistant parasites. |
| | | Although studies are underway, there are currently insufficient data to parameterize the model for migration. | Out-migration of infected individuals would promote the spread of malaria and drug resistance. |

| | | |
|---|---|---|
| No stochasticity | A deterministic framework was chosen to maintain flexibility and efficiency to rapidly explore a wide range of scenarios in accordance with the aims of this study. | In a stochastic framework, infections more likely to spontaneously eliminate when numbers are small. |
| Single infecting clone in each individual | To maintain simplicity. A model of multiple clones within a host would be far more complex than that presented here. | The mixing of multiple clones within a host may aid in the selection of fitter parasites through competition for resources. This would reduce the likelihood of elimination. |
| | Most infections in Cambodia are with a single clone,[4] although multiple clones are common in high transmission settings. | Alternatively, interbreeding between clones may dilute any drug resistance mutations in the population and thus increase the likelihood of elimination. |
| | It is not understood how multiple clones interact within a host and how this affects the transmission dynamics. | |
| No resistance emerging to piperaquine therefore no concomitant resistance to piperaquine and artemisinin. | Concomitant resistance to both an artemisinin and piperaquine has not been described. | Without resistance, piperaquine contributes more to the overall efficacy of ACT at the population level. The relative efficacy of ACT compared to atovaquone-proguanil will be greater than if partner drug resistance had been included. |
| | To preserve simplicity. Adding a third drug resistance phenotype would add greatly to the complexity of the model. | |

## References


1. Pacific WHOROftW: **Cambodia Health Situation and Trend.** Manila, Philippines: World Health Organization Regional Office for the Western Pacific; 2007.
2. Yeung S, Van Damme W, Socheat D, White NJ, Mills A: **Access to artemisinin combination therapy for malaria in remote areas of Cambodia.** *Malaria journal* 2008, **7:**96.
3. World Health Organization.: *Guidelines for the treatment of malaria.* 2nd edn. Geneva: World Health Organization; 2010.
4. Anderson TJ, Nair S, Nkhoma S, Williams JT, Imwong M, Yi P, Socheat D, Das D, Chotivanich K, Day NP, White NJ, Dondorp AM: **High heritability of malaria parasite clearance rate indicates a genetic basis for artemisinin resistance in western Cambodia.** *J Infect Dis* 2010, **201:**1326-1330.
