# Supplementary material for: The diminishing returns of atovaquone-proguanil for elimination of Plasmodium falciparum malaria: modelling mass drug administration and treatment
Source: Malar J. 2014 Sep 24;13:380. doi: 10.1186/1475-2875-13-380 (PMC4192368; doi:10.1186/1475-2875-13-380)
Supplement: Supplementary file 2 — Additional file 2: Table S2: Parameters. (PDF 308 KB) [file 12936_2014_3549_MOESM2_ESM.pdf]

Parameters

	Symbol	Description	Value	Source
**Population demographics**	N_0	Population size	1,000,000	[1]
	M	Birth rate = death rate	25.2/1000/year	[2]
**Prevalence of malaria in population**	p_{inf}	Mean proportion of population with Plasmodium falciparum parasites in peripheral blood at baseline	0.0 - 0.15 – 0.7	[3-7], expert opinion
	$sens_{Dx}$	Sensitivity of diagnostic test used to detect parasites in peripheral blood in field conditions	0.8	Expert opinion
	p_a	Proportion of malaria infections that are resistant to artesunate in 2013	0.0-0.1-0.5	Expert opinion
	p_b	Proportion of malaria infections that are resistant to atovaquone in 2013	0.0	Expert opinion
**Natural history of malaria infection**	Δ	Recovery rate from untreated infectious blood stage infection (nonimmune)	1/200 - 1/60 $days^{-1}$	[8-13]
	δ_R	Recovery rate from untreated infection (immune)	1/200 - 1/30 $days^{-1}$	[8-13]

	Γ	Rate of liver stage becoming blood stage	1/5 days^{-1}	[8-10]
	Σ	Rate of blood stage becoming gametocytes	1/15 days^{-1}	[14, 15]
	Amp	Amplitude of seasonal variation of transmission	0.34	Fitting
	Phi	Peak of seasonal variation of transmission	0.61	Fitting
	Ω	Duration of immunity to malaria	1 years	[16]
	$propRx_R$	Proportion of infected cases with immunity who are treated	0.1	[16]
	$propRx_N$	Proportion of infected cases without immunity who are treated	0.9	[16]
	para_sym:asym	the relative median parasite burdens in symptomatic vs asymptomatic individuals	100,000:1, <u>1:1000,</u> 1:1	Expert opinion
**Artemisinin monotherapy**	$start_a$	Year of introduction of artemisinin monotherapy	1975	Expert opinion
	$\tau = \tau_{ai1} = \tau_{ai2}$	Rate of starting artemisinin monotherapy	1/16 infected people per day	[17]

	$propRx_{am}$	Proportion of infected population who receive antimalarials	0.63	[17]
	$prop_a$	Proportion of antimalarials constituting artemisinin monotherapy	0.4	[17]
	adh_a	Proportion of infected population that take full 7 day course of artemisinin monotherapy	0.2	[17]
**ACT treatment**	$start_{ab}$	Year of introduction of ACT	2000	[18]
	$prop_{ab}$	Proportion of antimalarials constituting ACT	0.72	[19]
	adh_{ab}	Adherence to 3 day course of ACT	0.77-<u>0.92</u>-1.0	[17, 20-22]
	$start_{ab2}$	Year coverage with ACT treatment increased	2014	Expert opinion
	$\tau_{ab} = \tau_{ab1} = \tau_{ab2}$	Rate of reaching maximum coverage with ACT treatment	1/14 $days^{-1}$	[17]
	cov_{ab}	Maximum coverage with ACT for symptomatic cases	0.95	Expert opinion
	$propRx_{ab2}$	Proportion of infected population that take effective ACT after coverage increased	0.87	= $cov_{ab}*adh_{ab}$

**Atovaquone-proguanil treatment**	$start_{vg}$	Year of introduction of atovaquone-proguanil in place of ACT	2014	Expert opinion
	$prop_{vg}$	Proportion of antimalarials constituting atovaquone-proguanil following its' adoption instead of ACT for treatment of symptomatic cases	0.72	= $prop_{ab}$
	cov_{vg}	Maximum coverage with atovaquone-proguanil for treatment of symptomatic cases	0.95	= cov_{ab}
**Mass drug administration**	$\tau_{MDA1} = \tau_{MDA2}$	Rate of reaching maximum coverage with MDA	1/14 days^{-1}	Expert opinion
	cov_{MDA1}	Coverage of first MDA	0.8 - <u>0.95</u>	Expert opinion
	cov_{MDA2}	Coverage of second MDA	0.8 - <u>0.95</u>	Expert opinion
	T_{MDA2}	Time between first and second rounds of MDA	30 - 365 days	Expert opinion
	$dur_{i1} = dur_{i2}$	Duration of single round of MDA	14 days	Expert opinion
	adh_{vg}	Adherence to 3 day course of atovaquone-proguanil	0.88-<u>0.92</u>-1.0	[23-25]

	$propRx_{i1}$ = $propRx_{i2}$	Proportion that receive full 3 day course of MDA	0.87	= cov_{MDA1}*adh_{ab} or cov_{MDA2}*adh_{vg}
**Duration of efficacy against sensitive parasites (X)**	X_{ao}	Full course of artemisinin monotherapy	7 days	[26, 27]
	X_{ai}	Artemisinin as part of ACT (3 day course)	3 days	[26]
	X_b	Piperaquine	20 days	[28]
	Xg	Proguanil as part of atovaquone-proguanil	4 days	[29]
	Xv	Atovaquone	15 days	[30]
**Rates of clearance of drug sensitive infection (ν) by treatment**	c_{Broda}	Artemisinin vs non-infectious blood stage	1/7 $days^{-1}$	[31]
	c_{Iroda}	Artemisinin vs infectious blood stage	1/4$days^{-1}$	[32]
	C_{Irodab}, C_{Brodab}	Artemether-piperaquine vs infectious or non-infectious blood stage	1/3 $days^{-1}$	[31, 33, 34]

	c_{Brodb}	Piperaquine vs non-infectious blood stage	1/3 $days^{-1}$	[35]
	c_{Irodb}	Piperaquine vs infectious blood stage	1/21 $days^{-1}$	[33]
	c_{Lrodvg}	Atovaquone-proguanil vs liver stage	1/3 $days^{-1}$	[36]
	c_{Brodvg}	Atovaquone-proguanil vs non-infectious blood stage	1/3 $days^{-1}$	[30]
	c_{Irodvg}	Atovaquone-proguanil vs infectious blood stage	1/(4.5) $days^{-1}$	Unpublished data from Lee S
	c_{Lrodv}	Atovaquone vs liver stage	1/6 $days^{-1}$	[36]
	c_{Brodv}	Atovaquone vs non-infectious blood stage	1/3 $days^{-1}$	[30]
	c_{Irodv}	Atovaquone vs infectious blood stage	1/(4.5) $days^{-1}$	Unpublished data from Lee S
**Drug resistance**	P_{ra}	Proportion of infections that are resistant to artesunate at the time of first MDA	0.0-<u>0.1</u>-0.5	Expert opinion
	P_{rv}	Proportion of infections that are resistant to atovaquone	0.0	Expert opinion
	pct_{roda}	Parasite clearance time for artemisinin vs sensitive infections	30 hours	[37]

	pct_{rada}	Parasite clearance time for artemisinin vs resistant infections	83 hours	[37]
	ε_{rada}	Relative effectiveness of artemisinin against artemisinin resistant parasites	0.27	$= pct_{roda}/pct_{rada} *(1-p_{recra})$
	ε_{rbdb}	Relative effectiveness of piperaquine against resistant parasites	0.8	[35]
	ε_{rbdv}	Relative effectiveness of atovaquone against resistant parasites	0.01	[38]
	p_{rvdvg}	Proportion of infections treated with atovaquone-proguanil that develop atovaquone resistance	0.3	[38-40]
	acq_{rvdvg}	Rate of atovaquone resistance arising during treatment with atovaquone-proguanil	1/1000, 1/365, 1/10 $days^{-1}$	[38, 40]
	acq_{rvdv}	Rate of atovaquone resistance arising during treatment with atovaquone	1/10, 1/1, 1/0.5 $days^{-1}$	Varied to give $p_{rvdvg=0.3}$ as a model output
	$cost_{ra}$	Fitness cost of artemisinin resistance	0.0	[41]
	$cost_{rv}$	Fitness cost of atovaquone resistance	0.05	[42]
**Bed nets**	ρ	Degree of transmission reduction (the product of coverage and efficacy)	0.3	[43, 44]

tau_{bn}	Time to introduce bed nets	1 month	Expert opinion
cov_{bn}	Coverage with insecticide treated bed nets	0 or 0.75	Expert opinion
dur_{bn}	Duration of effectiveness of bed nets	2 years	Expert opinion

References

1. **General Population Census of Cambodia** [https://www.cia.gov/library/publications/the-world-factbook/geos/cb.html]
2. **The World Factbook. East & Southeast Asia: Cambodia** [https://www.cia.gov/library/publications/the-world-factbook/geos/cb.html]
3. Incardona S, Vong S, Chiv L, Lim P, Nhem S, Sem R, Khim N, Doung S, Mercereau-Puijalon O, Fandeur T: **Large-scale malaria survey in Cambodia: novel insights on species distribution and risk factors.** *Malaria journal* 2007, **6:**37.
4. Dysoley L, Kaneko A, Eto H, Mita T, Socheat D, Borkman A, Kobayakawa T: **Changing patterns of forest malaria among the mobile adult male population in Chumkiri District, Cambodia.** *Acta Trop* 2008, **106:**207-212.
5. Steenkeste N, Rogers WO, Okell L, Jeanne I, Incardona S, Duval L, Chy S, Hewitt S, Chou M, Socheat D, Babin FX, Ariey F, Rogier C: **Sub-microscopic malaria cases and mixed malaria infection in a remote area of high malaria endemicity in Rattanakiri province, Cambodia: implication for malaria elimination.** *Malar J* 2010, **9**.
6. Cook J, Speybroeck N, Sochanta T, Somony H, Sokny M, Claes F, Lemmens K, Theisen M, Soares IS, D'Alessandro U, Coosemans M, Erhart A: **Sero-epidemiological evaluation of changes in *Plasmodium falciparum* and *Plasmodium vivax* transmission patterns over the rainy season in Cambodia.** *Malar J* 2012, **11:**86.
7. Hoyer S, Nguon S, Kim S, Habib N, Khim N, Sum S, Christophel EM, Bjorge S, Thomson A, Kheng S, Chea N, Yok S, Top S, Ros S, Sophal U, Thompson MM, Mellor S, Ariey F, Witkowski B, Yeang C, Yeung S, Duong S, Newman RD, Menard D: **Focused Screening and Treatment (FSAT): a PCR-based strategy to detect malaria parasite carriers and contain drug resistant *P. falciparum*, Pailin, Cambodia.** *PLoS One* 2012, **7:**e45797.
8. Eyles DE, Young MD: **The duration of untreated or inadequately treated *Plasmodium falciparum* infections in the human host.** *Journal National Malaria Society* 1951, **10:**327-336.

9. Collins WE, Jeffery GM: **A retrospective examination of sporozoite- and trophozoite-induced infections with *Plasmodium falciparum*: development of parasitologic and clinical immunity during primary infection.** *The American journal of tropical medicine and hygiene* 1999, **61:**4-19.
10. Kitchen SF: **Falciparum Malaria.** In *Malariology.* Edited by Boyd MF. Philadelphia: W.B. Saunders Co.; 1949: 995-1016
11. Franks S, Koram KA, Wagner GE, Tetteh K, McGuinness D, Wheeler JG, Nkrumah F, Ranford-Cartwright L, Riley EM: **Frequent and persistent, asymptomatic *Plasmodium falciparum* infections in African infants, characterized by multilocus genotyping.** *The Journal of infectious diseases* 2001, **183:**796-804.
12. Bruce MC, Donnelly CA, Packer M, Lagog M, Gibson N, Narara A, Walliker D, Alpers MP, Day KP: **Age- and species-specific duration of infection in asymptomatic malaria infections in Papua New Guinea.** *Parasitology* 2000, **121 (Pt 3):**247-256.
13. Babiker HA, Abdel-Muhsin AM, Ranford-Cartwright LC, Satti G, Walliker D: **Characteristics of *Plasmodium falciparum* parasites that survive the lengthy dry season in eastern Sudan where malaria transmission is markedly seasonal.** *The American journal of tropical medicine and hygiene* 1998, **59:**582-590.
14. Thompson D: **A research into the production, life and death of crescents in malignant tertian malaria, in treated and untreated cases by an enumerative method. .** *Ann Trop Med Parasitol* 1911, **5:**57-85.
15. Jeffrey GM, Eyles DE: **Infectivity to mosquitoes of *Plasmodium falciparum* as related to gametocyte density and duration of infection. .** *Am J Trop Med Hyg* 1955, **4:**781-789.
16. Aguas R, Lourenco JM, Gomes MG, White LJ: **The impact of IPTi and IPTc interventions on malaria clinical burden - in silico perspectives.** *PLoS One* 2009, **4:**e6627.
17. Yeung S, Van Damme W, Socheat D, White NJ, Mills A: **Access to artemisinin combination therapy for malaria in remote areas of Cambodia.** *Malaria journal* 2008, **7:**96.
18. Ministry of Health National Centre for Parasitology EaMC: **Annual progress report of the National for Parasitology Entomology and Malaria Control Program 2009.** Phnom Penh: Ministry of Health, Cambodia; 2009.
19. Littrell M, Gatakaa H, Phok S, Allen H, Yeung S, Chuor CM, Dysoley L, Socheat D, Spiers A, White C, Shewchuk T, Chavasse D, O'Connell KA: **Case management of malaria fever in Cambodia: results from national anti-malarial outlet and household surveys.** *Malaria journal* 2011, **10:**328.
20. Naing C, Mak JW, Aung K, Wong JY: **Efficacy and safety of dihydroartemisinin-piperaquine for treatment of uncomplicated *Plasmodium falciparum* malaria in endemic countries: meta-analysis of randomised controlled studies.** *Trans R Soc Trop Med Hyg* 2013, **107:**65-73.
21. Zwang J, Ashley EA, Karema C, D'Alessandro U, Smithuis F, Dorsey G, Janssens B, Mayxay M, Newton P, Singhasivanon P, Stepniewska K, White NJ, Nosten F: **Safety and efficacy of dihydroartemisinin-piperaquine in falciparum malaria: a prospective multi-centre individual patient data analysis.** *PLoS One* 2009, **4:**e6358.

22. Smithuis F, Kyaw MK, Phe O, Aye KZ, Htet L, Barends M, Lindegardh N, Singtoroj T, Ashley E, Lwin S, Stepniewska K, White NJ: **Efficacy and effectiveness of dihydroartemisinin-piperaquine versus artesunate-mefloquine in falciparum malaria: an open-label randomised comparison.** *Lancet* 2006, **367:**2075-2085.
23. Cordel H, Cailhol J, Matheron S, Bloch M, Godineau N, Consigny PH, Gros H, Campa P, Bouree P, Fain O, Ralaimazava P, Bouchaud O: **Atovaquone-proguanil in the treatment of imported uncomplicated *Plasmodium falciparum* malaria: a prospective observational study of 553 cases.** *Malar J* 2013, **12:**399.
24. Blonde R, Naudin J, Bigirimana Z, Holvoet L, Fenneteau O, Vitoux C, Bourdon O, Angoulvant F, Lorrot M, D'Ortenzio E, Bourrillon A, Le Bras J, Matheron S, Faye A: **[Tolerance and efficacy of atovaquone-proguanil for the treatment of paediatric imported *Plasmodium falciparum* malaria in France: clinical practice in a university hospital in Paris].** *Arch Pediatr* 2008, **15:**245-252.
25. Bouchaud O, Muhlberger N, Parola P, Calleri G, Matteelli A, Peyerl-Hoffmann G, Mechai F, Gautret P, Clerinx J, Kremsner PG, Jelinek T, Kaiser A, Beltrame A, Schmid ML, Kern P, Probst M, Bartoloni A, Weinke T, Grobusch MP: **Therapy of uncomplicated falciparum malaria in Europe: MALTHER - a prospective observational multicentre study.** *Malar J* 2012, **11:**212.
26. White NJ: **Assessment of the pharmacodynamic properties of antimalarial drugs in vivo.** *Antimicrobial agents and chemotherapy* 1997, **41:**1413-1422.
27. Karbwang J, Na-Bangchang K, Congpoung K, Thanavibul A, Harinasuta T: **Pharmacokinetics of oral artesunate in thai patients with uncomplicated falciparum malaria.** *Clinical drug investigation* 1998, **15:**37-43.
28. Tarning J, Ashley EA, Lindegardh N, Stepniewska K, Phaiphun L, Day NP, McGready R, Ashton M, Nosten F, White NJ: **Population pharmacokinetics of piperaquine after two different treatment regimens with dihydroartemisinin-piperaquine in patients with *Plasmodium falciparum* malaria in Thailand.** *Antimicrobial agents and chemotherapy* 2008, **52:**1052-1061.
29. Wattanagoon Y, Taylor RB, Moody RR, Ochekpe NA, Looareesuwan S, White NJ: **Single dose pharmacokinetics of proguanil and its metabolites in healthy subjects.** *British journal of clinical pharmacology* 1987, **24:**775-780.
30. van Vugt M, Leonardi E, Phaipun L, Slight T, Thway KL, McGready R, Brockman A, Villegas L, Looareesuwan S, White NJ, Nosten F: **Treatment of uncomplicated multidrug-resistant falciparum malaria with artesunate-atovaquone-proguanil.** *Clinical infectious diseases : an official publication of the Infectious Diseases Society of America* 2002, **35:**1498-1504.
31. Adjuik M, Babiker A, Garner P, Olliaro P, Taylor W, White N: **Artesunate combinations for treatment of malaria: meta-analysis.** *Lancet* 2004, **363:**9-17.
32. Maude RJ, Pontavornpinyo W, Saralamba S, Aguas R, Yeung S, Dondorp AM, Day NP, White NJ, White LJ: **The last man standing is the most resistant: eliminating artemisinin-resistant malaria in Cambodia.** *Malar J* 2009, **8:**31.
33. Myint HY, Ashley EA, Day NP, Nosten F, White NJ: **Efficacy and safety of dihydroartemisinin-piperaquine.** *Transactions of the Royal Society of Tropical Medicine and Hygiene* 2007, **101:**858-866.

34. Janssens B, van Herp M, Goubert L, Chan S, Uong S, Nong S, Socheat D, Brockman A, Ashley EA, Van Damme W: **A randomized open study to assess the efficacy and tolerability of dihydroartemisinin-piperaquine for the treatment of uncomplicated falciparum malaria in Cambodia.** *Tropical medicine & international health : TM & IH* 2007, **12:**251-259.
35. Chen L, Qu FY, Zhou YC: **Field observations on the antimalarial piperaquine.** *Chinese medical journal* 1982, **95:**281-286.
36. Berman JD, Nielsen R, Chulay JD, Dowler M, Kain KC, Kester KE, Williams J, Whelen AC, Shmuklarsky MJ: **Causal prophylactic efficacy of atovaquone-proguanil (Malarone) in a human challenge model.** *Transactions of the Royal Society of Tropical Medicine and Hygiene* 2001, **95:**429-432.
37. Dondorp AM, Nosten F, Yi P, Das D, Phyo AP, Tarning J, Lwin KM, Ariey F, Hanpithakpong W, Lee SJ, Ringwald P, Silamut K, Imwong M, Chotivanich K, Lim P, Herdman T, An SS, Yeung S, Singhasivanon P, Day NP, Lindegardh N, Socheat D, White NJ: **Artemisinin resistance in Plasmodium falciparum malaria.** *N Engl J Med* 2009, **361:**455-467.
38. Looareesuwan S, Viravan C, Webster HK, Kyle DE, Hutchinson DB, Canfield CJ: **Clinical studies of atovaquone, alone or in combination with other antimalarial drugs, for treatment of acute uncomplicated malaria in Thailand.** *Am J Trop Med Hyg* 1996, **54:**62-66.
39. Chiodini PL, Conlon CP, Hutchinson DB, Farquhar JA, Hall AP, Peto TE, Birley H, Warrell DA: **Evaluation of atovaquone in the treatment of patients with uncomplicated Plasmodium falciparum malaria.** *J Antimicrob Chemother* 1995, **36:**1073-1078.
40. Looareesuwan S, Chulay JD, Canfield CJ, Hutchinson DB: **Malarone (atovaquone and proguanil hydrochloride): a review of its clinical development for treatment of malaria. Malarone Clinical Trials Study Group.** *Am J Trop Med Hyg* 1999, **60:**533-541.
41. Walliker D, Hunt P, Babiker H: **Fitness of drug-resistant malaria parasites.** *Acta tropica* 2005, **94:**251-259.
42. Peters JM, Chen N, Gatton M, Korsinczky M, Fowler EV, Manzetti S, Saul A, Cheng Q: **Mutations in cytochrome b resulting in atovaquone resistance are associated with loss of fitness in Plasmodium falciparum.** *Antimicrob Agents Chemother* 2002, **46:**2435-2441.
43. Dolan G, ter Kuile FO, Jacoutot V, White NJ, Luxemburger C, Malankirii L, Chongsuphajaisiddhi T, Nosten F: **Bed nets for the prevention of malaria and anaemia in pregnancy.** *Transactions of the Royal Society of Tropical Medicine and Hygiene* 1993, **87:**620-626.
44. Sochantha T, Hewitt S, Nguon C, Okell L, Alexander N, Yeung S, Vannara H, Rowland M, Socheat D: **Insecticide-treated bednets for the prevention of Plasmodium falciparum malaria in Cambodia: a cluster-randomized trial.** *Tropical medicine & international health : TM & IH* 2006, **11:**1166-1177.
